# Pentoxifylline enhances antioxidative capability and promotes mitochondrial biogenesis for improving age-related behavioral deficits

**DOI:** 10.18632/aging.104155

**Published:** 2020-11-20

**Authors:** Yu Wang, Yunxiao Kang, Chunxiao Qi, Tianyun Zhang, Hui Zhao, Xiaoming Ji, Wensheng Yan, Yuanxiang Huang, Rui Cui, Guoliang Zhang, Geming Shi

**Affiliations:** 1 Department of Neurobiology, Hebei Medical University, Shijiazhuang 050017, China; 2 Department of Anatomy, Hebei Medical University, Shijiazhuang 050017, China; 3 Department of Sports Medicine, Hebei Sport University, Shijiazhuang 050017, China; 4 Neuroscience Research Center, Hebei Medical University, Shijiazhuang 050017, China; 5 Hebei Key Laboratory of Neurodegenerative Disease Mechanism, Hebei Medical University, Shijiazhuang 050017, China

**Keywords:** pentoxifylline, antioxidative capability, mitochondrial biogenesis, aged rats

## Abstract

Pentoxifylline (PTX) is a non-specific phosphodiesterase inhibitor with pleiotropic effects that is routinely used to treat peripheral vascular disease. In this study, we tested whether PTX could also counteract the detrimental effects of aging in the brain. To accomplish that, we treated aged rats with PTX and measured resulting behavioral alterations as well as changes in dopaminergic neurochemical levels, oxidative balance markers, mitochondrial function, nuclear factor erythroid 2-related factor 2 (Nrf2), peroxisome proliferator activated receptor-gamma coactivator 1-alpha (PGC-1α) and downstream gene expression, and cyclic adenosine monophosphate (cAMP) content in the brain. The results demonstrated that PTX improved motor and cognitive deficits and restored levels of dopamine and its metabolites in the brains of aged rats. PTX also reduced malondialdehyde levels and increased the GSH/GSSG ratio, mitochondrial ATP, nuclear Nrf2, and cAMP levels, and upregulated PGC-1α, nuclear respiratory factor 1, and mitochondrial transcription factor A expression in the substantia nigra and hippocampus of aged rats. Thus, increased nuclear Nrf2 levels and upregulation of PGC-1α, which enhance antioxidative capability and promote mitochondrial biogenesis, may be responsible for PTX-induced amelioration of behavioral deficits in aged rats.

## INTRODUCTION

The proportion of the population that is 60 years old or above is rising globally, and increase in life expectancy accelerates aging of populations [1]. The inevitable and irreversible process of aging is a key element in most neurodegenerative diseases and is characterized by physical deterioration, which impacts both lifespan and quality of life [2]. Most neurodegenerative diseases, such as Parkinson’s disease and Alzheimer’s disease, are characterized by deficits in motor and cognitive functions that are associated with large socioeconomic and personal burdens [3]. As the elderly population increases, the number of individuals living with age-related diseases will grow; effective preventive measures and therapies are therefore urgently needed.

Aging is a complex process. The generation of reactive oxygen species (ROS) and biological responses to ROS are increasingly recognized as crucial factors impacting longevity [4]. While ROS can exert beneficial effects on physiological functions such as cell signaling and homeostasis [5], excessive ROS levels have detrimental effects on mitochondrial function, calcium signaling, and other processes [6]. Increased ROS levels result in oxidative damage-induced mitochondrial dysfunction and potentially accelerate aging and the onset of age-related diseases [7, 8]. Under normal physiological conditions, the mitochondrial respiratory chain (MRC) generates a proton motive force that sustains mitochondrial membrane potential and is used to synthesize the high-energy compound adenosine triphosphate (ATP) along with a small amount of ROS. In pathological conditions, overproduction of ROS impairs mitochondrial function by reducing MRC component synthesis, decreasing ATP production, and depolarizing the mitochondrial membrane potential, which induces severe oxidative stress [9]. Mitochondrial dysfunctions, such as excessive ROS levels, low ATP levels, and reduced MRC complex activity, are more common in the aged brain [10–12]. Thus, reducing oxidative damage to mitochondria and reversing mitochondrial dysfunctions during the aging process might help maintain normal brain function in advanced age.

Nuclear factor erythroid 2-related factor 2 (Nrf2) is a transcription factor that regulates the expression of antioxidant proteins. It protects cells against oxidative, inflammatory, and metabolic stresses [13, 14] by controlling the expression of a series of detoxification and antioxidant enzymes through antioxidant response element (ARE) [15]. Activation of the Nrf2-ARE pathway protects cells against insults from oxidative stress [15], and disruption of the Nrf2-ARE pathway leads to increased oxidative damage [16]. Suppression of Nrf2 signaling is a crucial contributor to the premature aging phenotype of Hutchinson-Gilford progeria syndrome, a rare fatal premature aging disorder [17]. In addition, emerging evidence indicates that Nrf2 modulates mitochondrial biogenesis, particularly under stress conditions [18]. Mitochondrial biogenesis is a complex process during which new mitochondria are formed from preexisting mitochondria in the cells [19] to maintain mitochondrial functions and regulate antioxidant defense [20], and it is primarily regulated by peroxisome proliferator activated receptor-gamma (PPARγ) coactivator 1-alpha (PGC-1α). In addition to regulating mitochondrial biogenesis by promoting expression of nuclear respiratory factor 1 (NRF-1) and mitochondrial transcription factor A (TFAM), PGC-1α activates the antioxidant system and increases levels of ROS-detoxifying enzymes under high-ROS conditions [20, 21]. Upregulation of PGC-1α and activation of Nrf2 have therefore been proposed as potential early preventive measures to combat aging and age-related disorders [22, 23].

Pentoxifylline (PTX) is a non-specific phosphodiesterase inhibitor that is routinely used to treat peripheral vascular diseases and cerebrovascular diseases [24]. Furthermore, as a modulator of intracellular 3′-5′-cyclic adenosine monophosphate (cAMP) signaling pathways [25], it was found that PTX is able to modulate cellular oxidative stress [26, 27] and mitochondrial biogenesis [28] against nonalcoholic steatohepatitis [29], major depressive disorder [30], and cognitive dysfunction [31]. Because oxidative damage and mitochondrial dysfunction are also involved in aging and aging-related neurodegenerative diseases, PTX might serve as a potential intervention to inhibit aging-related processes. In this study, we therefore examined the effects of PTX treatment on antioxidative ability and mitochondrial biogenesis in aged rats by measuring Nrf2, PGC-1α, and PGC-1α-downstream gene expression levels, oxidative balance status, mitochondrial function, and behavioral parameters. We found that PTX treatment enhanced antioxidative capability, promoted mitochondrial biogenesis, and improved behavioral deficits in aged rats.

## RESULTS

### PTX treatment ameliorated motor behavior deficits in aged rats

**Figure 1:**
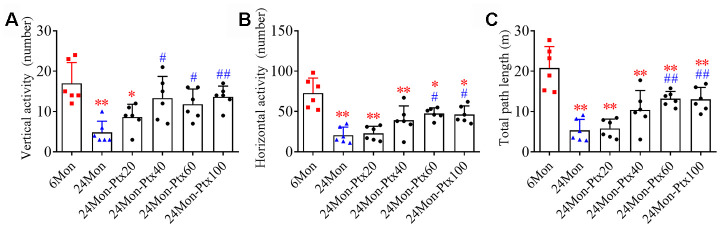
**Effects of PTX treatment on motor behavior deficits in aged rats.** (**A**) Effects of PTX treatment on vertical activity. (**B**) Effects of PTX treatment on horizontal activity. (**C**) Effects of PTX treatment on total path length. Data are expressed as the mean ± S.D. (n=6 rats/group). *P<0.05 versus 6Mon rats, **P<0.01 versus 6Mon rats, ^#^*P*<0.05 versus 24Mon rats, ^##^*P*<0.01 versus 24Mon rats.

We first performed an open field test to observe changes in motor behavior in 6-month-old rats (6Mon), 24-month-old rats (24Mon) and 24-month-old rats treated with PTX at 20 mg/kg (24Mon-Ptx20), 40 mg/kg (24Mon-Ptx40), 60 mg/kg (24Mon-Ptx60), or 100 mg/kg (24Mon-Ptx100). Vertical activity (Figure 1A, *P*<0.01), horizontal activity (Figure 1B, *P*<0.01), and total path length (Figure 1C, *P*<0.01) differed among the experimental groups. Post hoc tests revealed that vertical activity, horizontal activity, and total path length were significantly decreased in the 24Mon group compared to the 6Mon group (*P*<0.01). All three behavioral parameters were significantly increased in the 24Mon-Ptx60 (vertical activity and horizontal activity: *P*<0.05; total path length: *P*<0.01) and 24Mon-Ptx100 groups (horizontal activity: *P*<0.05; vertical activity and total path length: *P*<0.01) compared to the 24Mon group. Among the two lower-dose PTX groups (20 and 40 mg/kg), only vertical activity was increased in the 24Mon-Ptx40 group, indicating that higher PTX doses (60 and 100 mg/kg) were more effective.

### PTX treatment improved cognitive function in aged rats

**Figure 2:**
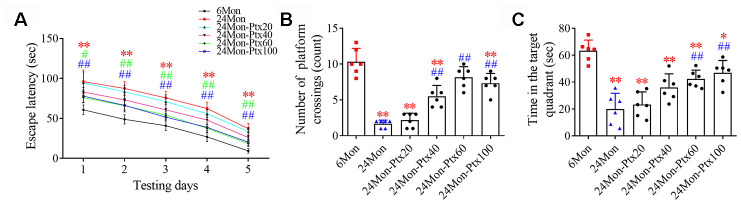
**Effects of PTX treatment on cognitive function in aged rats.** (**A**) Effects of PTX treatment on the escape latency of aged rats. (**B**) Effects of PTX treatment on the number of platform crossings. (**C**) Effects of PTX treatment on time spent in the target quadrant. Data are expressed as the mean ± S.D. (n=6 rats/group). *P<0.05 versus 6Mon rats, **P<0.01 versus 6Mon rats, ^#^*P*<0.05 versus 24Mon rats, ^##^*P*<0.01 versus 24Mon rats.

Next, the effects of PTX treatment on spatial learning and memory in aged rats were examined using a water maze test. Differences in escape latency (Figure 2A, *P*<0.01), number of platform crossings (Figure 2B, *P*<0.01), and time in the target quadrant (Figure 2C, *P*<0.01) were found among the experimental groups. Post hoc analysis showed that the 24Mon group exhibited a longer escape latency to reach the platform (*P*<0.01), fewer platform crossings (*P*<0.01), and less time in the target quadrant (*P*<0.01) compared to the 6Mon group. Administration of 60 or 100 mg/kg of PTX shortened escape latency to reach the platform (1d: 60 mg/kg, *P*<0.05; 100 mg/kg, *P*<0.01. 2-5d: *P*<0.01) and significantly increased the number of platform crossings (*P*<0.01) and time spent in the target quadrant (*P*<0.01) in aged rats. The only difference in these behavioral parameters between the 24Mon group and the 24Mon-Ptx20 and 24Mon-Ptx40 groups was an increased number of platform crossings in the 24Mon-Ptx40 group (*P*<0.01). Administration of 60 or 100 mg/kg PTX therefore improved spatial learning and memory capabilities in aged rats.

### PTX treatment increased dopaminergic neurochemical levels in the brains of aged rats

**Figure 3:**
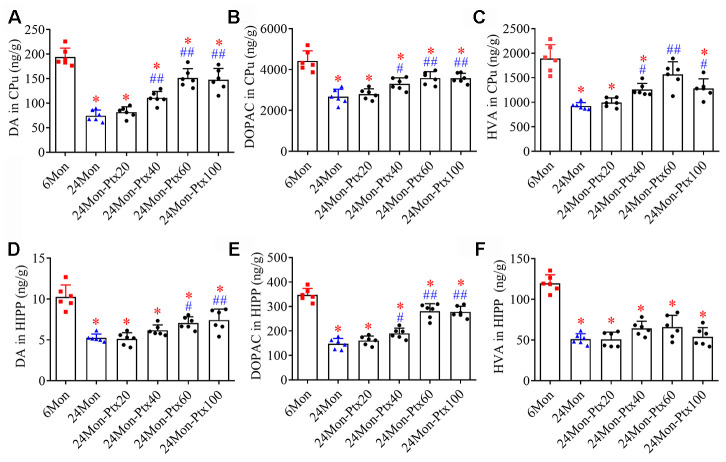
**Effects of PTX treatment on dopamine and its metabolites in the aged rat brain.** (**A**–**C**) Effects of PTX treatment on DA, DOPAC, and HVA in the CPu of aged rats. (**D**–**F**) Effects of PTX treatment on DA, DOPAC, and HVA in the HIPP of aged rats. Data are expressed as the mean ± S.D. (n=6 rats/group). *P<0.01 versus 6Mon rats, ^#^*P*<0.05 versus 24Mon rats, ^##^*P*<0.01 versus 24Mon rats.

Because the dopaminergic system is important for motor behavior and cognitive function, we next assessed the effects of PTX treatment on levels of dopamine (DA) and its metabolites 3,4-dihydroxyphenylacetic acid (DOPAC) and homovanillic acid (HVA) in the brains of aged rats. DA and metabolite levels were measured specifically in the hippocampus (HIPP) and the caudate-putamen (CPu), a brain region densely innervated by dopaminergic projections arising from the substantia nigra (SN). DA, DOPAC, and HVA levels differed among the experimental groups (Figure 3A–3C, CPu; Figure 3D–3F, HIPP. *P*<0.01). Post hoc tests revealed that DA, DOPAC, and HVA levels were significantly reduced in the CPu and HIPP of the 24Mon group relative to the 6Mon group (*P*<0.01) and were increased in the 24Mon-Ptx40 (CPu: DA, *P*<0.01; DOPAC, HVA, *P*<0.05. HIPP: DOPAC, *P*<0.05), 24Mon-Ptx60 (CPu: DA, DOPAC, HVA, *P*<0.01. HIPP: DA, *P*<0.05; DOPAC, *P*<0.01), and 24Mon-Ptx100 groups (CPu: DA, DOPAC, *P*<0.01; HVA, *P*<0.05. HIPP: DA, DOPAC, *P*<0.01) relative to the 24Mon group. PTX treatment at 60 or 100 mg/kg thus elevated dopaminergic neurochemical levels in the brains of aged rats.

### PTX treatment improved brain oxidative balance in aged rats

**Figure 4:**
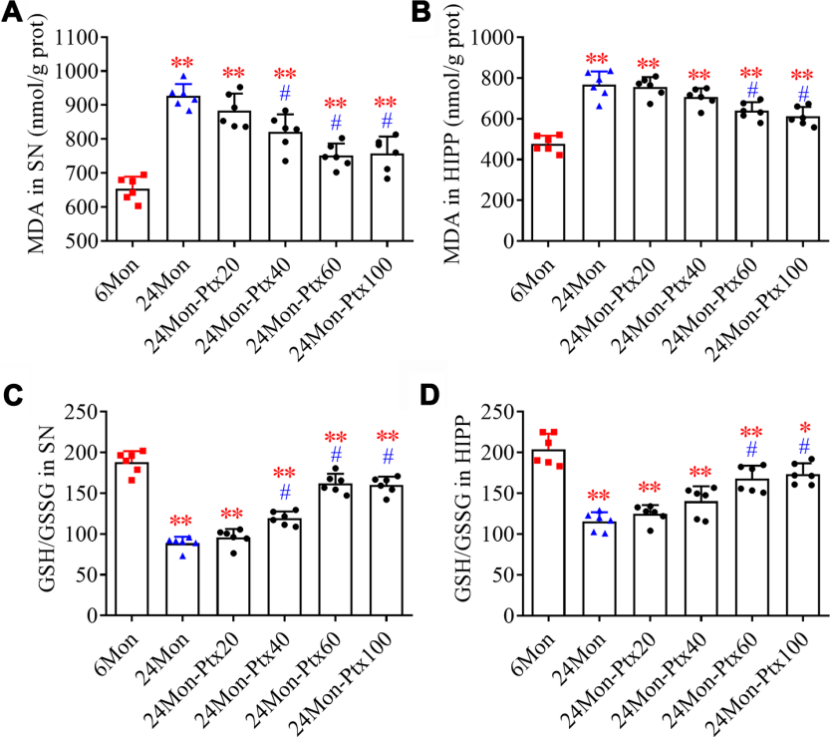
**Effects of PTX treatment on brain oxidative balance in aged rats.** (**A**, **B**) Effects of PTX treatment on MDA levels in the SN and HIPP of aged rats. (**C**, **D**) Effects of PTX treatment on GSH/GSSG ratio in the SN and HIPP of aged rats. Data are expressed as the mean ± S.D. (n=6 rats/group). *P<0.05 versus 6Mon rats, **P<0.01 versus 6Mon rats, ^#^*P*<0.01 versus 24Mon rats.

Malondialdehyde (MDA) levels and GSH/GSSG ratio, two important biomarkers of oxidative processes, were measured to assess oxidative balance in PTX-treated aged rats. MDA levels (Figure 4A, SN; Figure 4B, HIPP. *P*<0.01) and GSH/GSSG ratio (Figure 4C, SN; Figure 4D, HIPP. *P*<0.01) differed among the experimental groups. Post hoc tests revealed increased MDA levels and a decreased GSH/GSSG ratio in the SN and HIPP of the 24Mon group compared to the 6Mon group (*P*<0.01). In addition, MDA levels were reduced and the GSH/GSSG ratio was increased in the SN of the 24Mon-Ptx40, 24Mon-Ptx60, and 24Mon-Ptx100 groups, as well as in the HIPP of the 24Mon-Ptx60 and 24Mon-Ptx100 groups, compared to 24Mon rats (*P*<0.01). PTX at 60 or 100 mg/kg therefore improved oxidative balance in the SN and HIPP of aged rats.

### PTX administration increases mitochondrial ATP levels and mitochondrial complex activity in the aged rat brain

**Figure 5:**
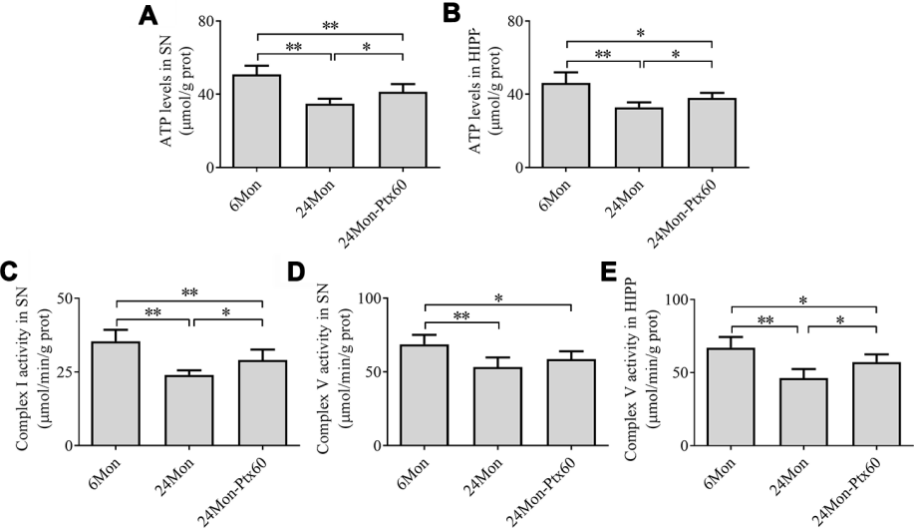
**Effects of PTX treatment on brain mitochondrial ATP levels and complex activity in aged rats.** (**A**) ATP levels in the SN. (**B**) ATP levels in the HIPP. (**C**) Mitochondrial complex I activity in the SN. (**D**, **E**) Mitochondrial complex V activity in the SN and HIPP. Data are expressed as the mean ± S.D. (n=6 rats/group). *P<0.05, **P<0.01.

Next, we examined ATP levels and mitochondrial complex activity in aged rats treated with 60 mg/kg of PTX, which was selected as the optimal dose based on the findings described above. Group differences in mitochondrial ATP levels in the SN (Figure 5A, *P*<0.01) and HIPP (Figure 5B, *P*<0.01) differed among the 6Mon, 24Mon, and 24Mon-Ptx60 groups. Post hoc tests indicated that mitochondrial ATP levels decreased by 31.5% in the SN and 28.6% in the HIPP of the 24Mon group relative to the 6Mon group (*P*<0.01). In addition, mitochondrial ATP levels increased by 18.4% in the SN and 15.2% in the HIPP of the 24Mon-Ptx60 group compared to the 24Mon group (*P*<0.05).

Of the five mitochondrial respiratory chain complexes, significant differences in complex I (Figure 5C) and complex V activity in the SN (Figure 5D) and HIPP (Figure 5E) were identified among the experimental groups (*P*<0.01). Post hoc tests revealed a 32.23% reduction in complex I activity in the SN, as well as decreases in complex V activity of 22.4% in the SN and 31.0% in the HIPP, in the 24Mon group relative to the 6Mon group (*P*<0.01). Additionally complex I activity in the SN increased by 21.1% (*P*<0.05) and complex V activity in the HIPP increased by 23.5% (*P*<0.05) in the 24Mon-Ptx60 group compared to the 24Mon group. No significant differences were detected in mitochondrial complex II, III, or IV activity among the experimental groups (Table 1).

**Table 1:** Effects of PTX treatment on mitochondrial complex activity (μmol/min/g protein).

**Enzyme**	**SN**			**HIPP**		
	**6Mon**	**24Mon**	**24Mon-Ptx60**	**6Mon**	**24Mon**	**24Mon-Ptx60**
complex I	35.43±3.89	24.01±1.54^*^	29.08±3.54^*Δ^	22.83±4.29	21.34±3.09	20.93±2.90
complex II	24.60±2.84	25.32±4.07	23.24±3.13	28.80±3.94	28.62±4.18	29.63±3.59
complex III	35.69±4.29	35.25±2.32	36.05±4.68	32.95±4.57	32.15±2.98	33.32±3.60
complex IV	18.70±3.11	18.04±2.40	20.25±3.62	14.10±3.42	12.66±3.33	13.08±3.74
complex V	68.69±6.44	53.30±6.49^*^	58.62±5.31^#^	67.06±7.30	46.30±6.22^*^	57.20±5.27^#Δ^

^*^P<0.01 versus 6Mon group, ^#^*P*<0.05 versus 6Mon group, ^Δ^*P*<0.01 versus 24Mon group.

### PTX treatment increased Nrf2 levels in the SN and HIPP of aged rats

**Figure 6:**
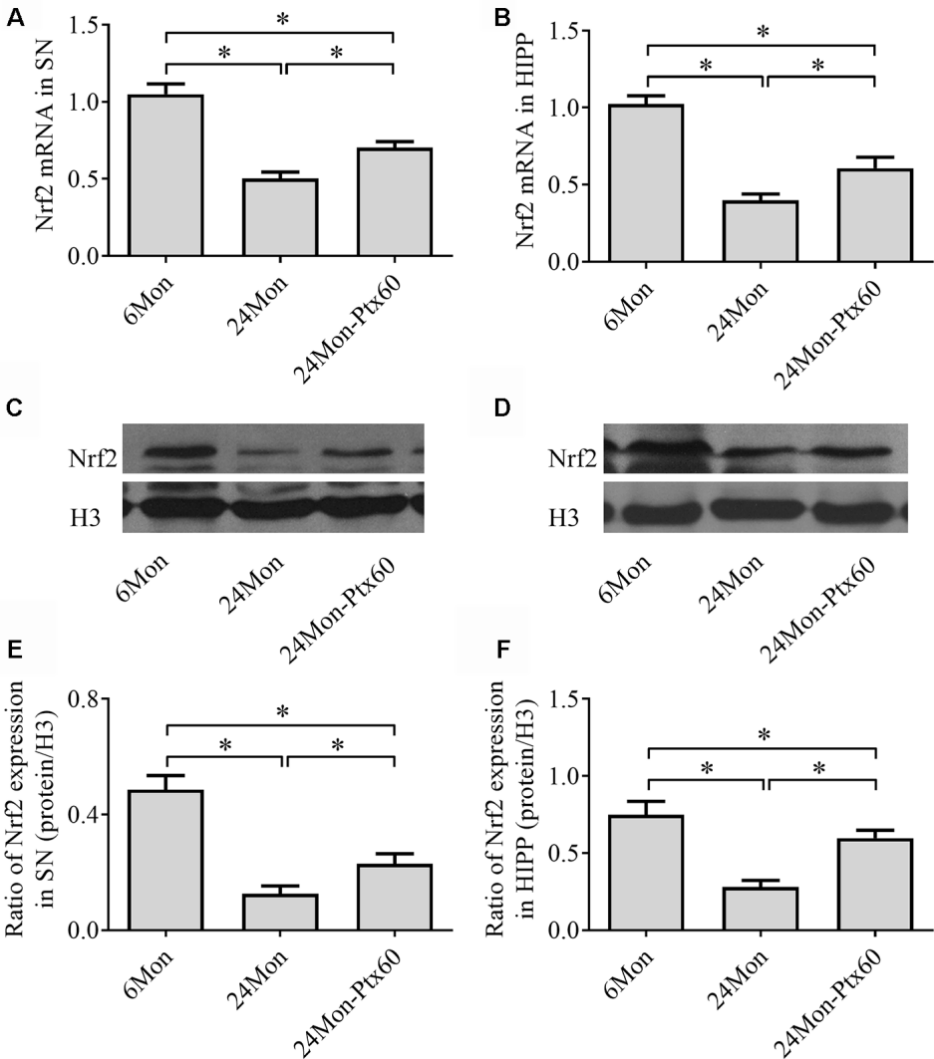
**Effects of PTX treatment on Nrf2 expression in the aged rat brain.** (**A**, **B**) *Nrf2* mRNA levels in the SN and HIPP were calculated using the 2^-ΔΔCt^ method. *GAPDH* was used as an internal control. (**C**, **D**) Representative Western blots of Nrf2 protein levels in the SN and HIPP. (**E**, **F**) Nrf2 protein levels in the SN and HIPP were quantified relative to H3 band density. Data are expressed as the mean ± S.D. (n=6 rats/group). *P<0.01.

Because Nrf2 plays a role in antioxidative defense systems and because PTX treatment altered oxidative balance in aged rats, we next examined the effects of PTX on Nrf2 levels in the SN and HIPP of aged rats. Nrf2 mRNA expression (Figure 6A, 6B, *P*<0.01) and protein levels in cell nuclei (Figure 6C–6F, *P*<0.01) differed significantly among the experimental groups in the SN and HIPP. As shown in Figure 6, Nrf2 mRNA and intranuclear Nrf2 protein levels were lower in the SN and HIPP of 24Mon rats than in 6Mon rats (*P*<0.01), while rats in the 24Mon-Ptx60 group had higher Nrf2 mRNA and intranuclear Nrf2 protein levels in the SN and HIPP than rats in the 24Mon group (*P*<0.01). Higher nuclear Nrf2 levels in PTX-treated aged rats indicate increased Nrf2 activation; PTX administration thus increased activated Nrf2 levels in the aged rat brain.

### PTX treatment upregulated PGC-1α and downstream gene expression in the aged rat brain

**Figure 7:**
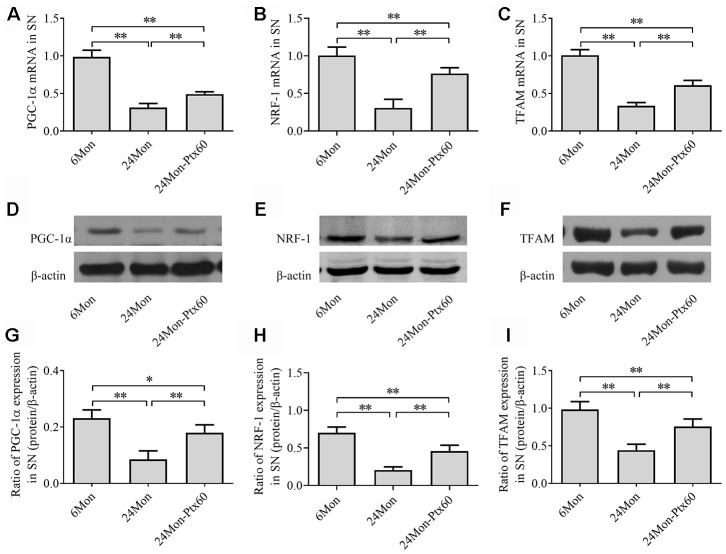
**Effects of PTX treatment on mitochondrial biogenesis in the SN in aged rats.** (**A**–**C**) *PGC-1α*, *NRF-1*, and *TFAM* mRNA levels were detected by qPCR. (**D**–**F**) Representative Western blots of PGC-1α, NRF-1, and TFAM protein levels. (**G**–**I**) PGC-1α, NRF-1, and TFAM protein levels were quantified relative to β-actin band density. Data are expressed as the mean ± S.D. (n=6 rats/group). *P<0.05, **P<0.01.

**Figure 8:**
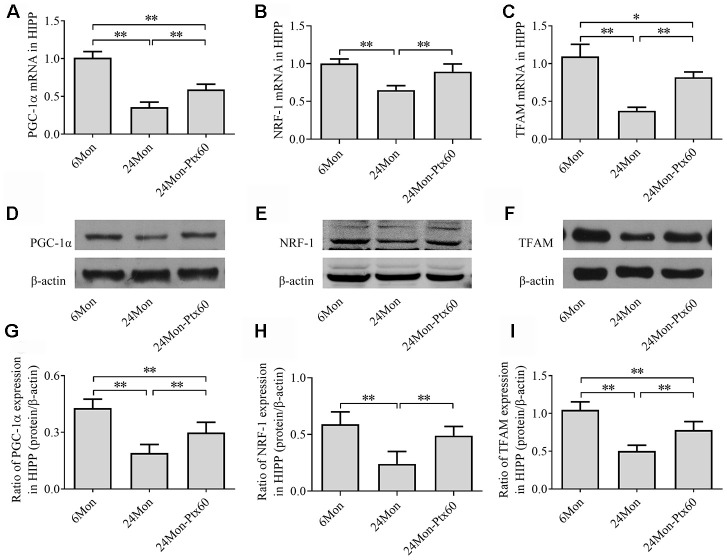
**Effects of PTX treatment on mitochondrial biogenesis in the HIPP in aged rats.** (**A**–**C**) *PGC-1α*, *NRF-1*, and *TFAM* mRNA levels were detected by qPCR. (**D**–**F**) Representative Western blots of PGC-1α, NRF-1, and TFAM protein levels. (**G**–**I**) PGC-1α, NRF-1, and TFAM protein levels were quantified relative to β-actin band density. Data are expressed as the mean ± S.D. (n=6 rats/group). *P<0.05, **P<0.01.

Because PGC-1α regulates mitochondrial biogenesis, and because PTX treatment altered mitochondrial function in aged rats, we next examined the effects of PTX administration on PGC-1α, NRF-1, and TFAM levels in the SN and HIPP of aged rats. PGC-1α, NRF-1, and TFAM mRNA expression and protein levels differed in both the SN and HIPP among the experimental groups (SN: Figure 7A–7I; HIPP: Figure 8A–8I. *P*<0.01). Post hoc analysis revealed that PGC-1α, NRF-1, and TFAM mRNA and protein expression was significantly downregulated in the 24Mon group compared to the 6Mon group (*P*<0.01) and upregulated in the 24Mon-Ptx60 group compared to the 24Mon group (*P*<0.01). PTX administration thus increased PGC-1α, NRF-1, and TFAM mRNA and protein expression in the brains of aged rats, indicating that PTX treatment promotes mitochondrial biogenesis during aging.

### PTX treatment increased mitochondrial content in the aged rat brain

**Figure 9:**
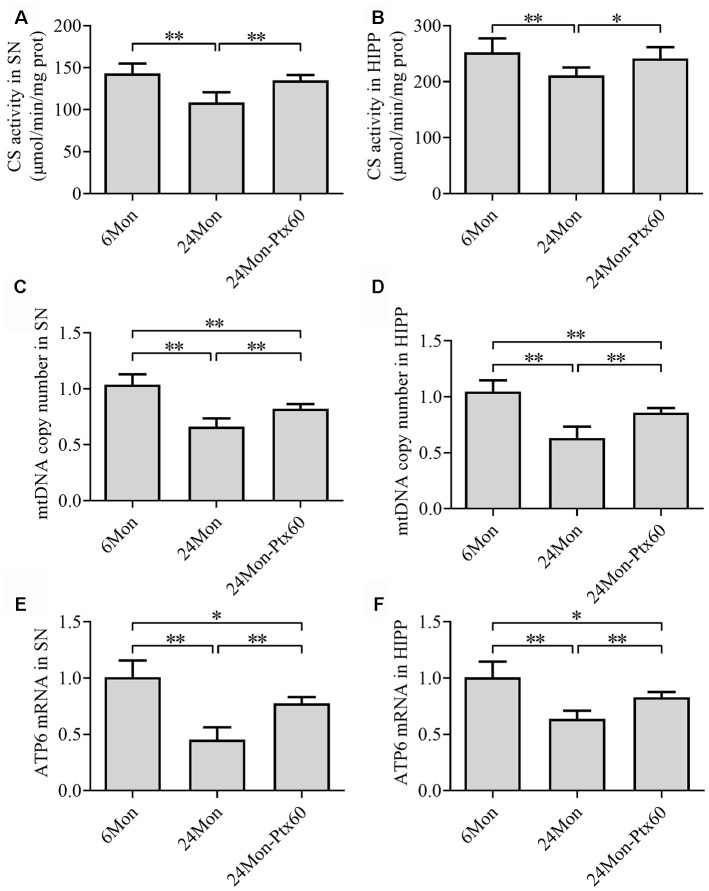
**Effects of PTX treatment on mitochondrial content in the aged rat brain.** (**A**, **B**) CS activity in the SN and HIPP. (**C**, **D**) mtDNA copy number in the SN and HIPP. (**E**, **F**) *ATP6* mRNA levels in the SN and HIPP. Data are expressed as the mean ± S.D. (n=6 rats/group). *P<0.05, **P<0.01.

To determine whether PTX-induced increases in PGC-1α/NRF-1/TFAM expression in the aged rat brain resulted in changes in mitochondrial content, we measured citrate synthase (CS) activity, mitochondrial DNA (mtDNA) copy number, and mtDNA-encoded subunit *ATP6* mRNA levels. CS activity, mtDNA copy number, and *ATP6* mRNA levels in the SN and HIPP differed significantly among the experimental groups (Figure 9A–9F, *P*<0.01). CS activity, mtDNA copy number, and *ATP6* mRNA levels were reduced by 24.1%, 33.3%, and 54.6%, respectively, in the SN and by 16.3%, 36.9%, and 36.1%, respectively, in the HIPP of the 24Mon group compared to the 6Mon group (*P*<0.01). In addition, CS activity, mtDNA copy number, and *ATP6* mRNA levels increased by 24.3%, 22.8%, and 70.9%, respectively, in the SN (*P*<0.01) and by 14.4%, 35.4%, and 29.7%, respectively, in the HIPP (CS, *P*<0.05; mtDNA copy number and *ATP6* mRNA, *P*<0.01) of the 24Mon-Ptx60 group compared to the 24Mon group.

### PTX treatment increased cAMP content in the aged rat brain

**Figure 10:**
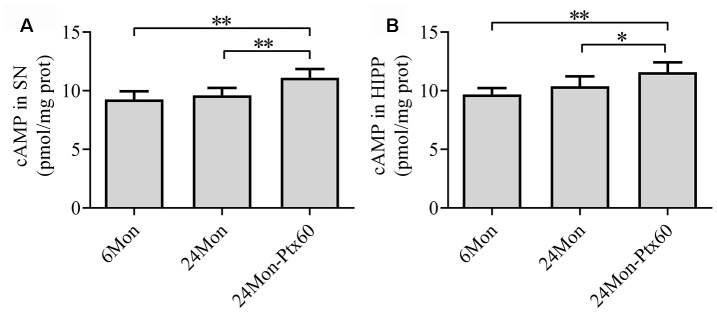
**Effects of PTX treatment on cAMP content in the aged rat brain.** (**A**) cAMP content in the SN. (**B**) cAMP content in the HIPP. Data are expressed as the mean ± S.D. (n=6 rats/group). *P<0.05, **P<0.01.

To examine a potential mechanism by which PTX might counteract the detrimental effects of aging in the brain, we measured cAMP content in the SN and HIPP in aged rats. cAMP content differed among the experimental groups in both the SN and HIPP (Figure 10A, 10B, *P*<0.01). cAMP content increased by 12% in the SN and 14.7% in the HIPP of the 24Mon-Ptx60 group compared to the 24Mon group (SN, *P*<0.01; HIPP, *P*<0.05).

## DISCUSSION

In this study, we identified several beneficial effects of PTX treatment in the aging brain. PTX treatment significantly ameliorated motor behavior and cognitive deficits, and restored brain levels of DA and its metabolites DOPAC and HVA, in aged rats. Reduced MDA levels, an elevated GSH/GSSG ratio, increased mitochondrial ATP levels, and increased mitochondrial complex I and V activity in the brains of PTX-treated aged rats demonstrated that PTX improved oxidative balance and reversed mitochondrial dysfunction. Increased Nrf2 activation, upregulation of PGC-1α/NRF-1/TFAM, and increases in CS activity, mtDNA copy number, and *ATP6* mRNA levels indicated that PTX treatment enhanced antioxidative ability and promoted mitochondrial biogenesis, possibly by increasing cAMP content. PTX administration thus effectively counteracted several detrimental aging-related processes in the brain largely by enhancing antioxidative capability and promoting mitochondrial biogenesis.

Motor abilities and cognitive function decline during normal aging [32, 33], and these declines are associated with reductions in dopaminergic neuron activity [34]. The activity of dopaminergic neurons in the SN decreases by about 5-10% per decade during aging [35, 36], leading to reduced levels of DA and its metabolites in the brain and contributing to decreases in locomotor activity [37] and cognitive function [33]. Consistent with previous findings, we observed deficits in motor behavior and cognitive capability, as well as reductions in brain dopaminergic neurochemical levels, in aged rats in this study. Reduced vertical activity, horizontal activity, and total path length, as well as longer escape latency to reach the platform, fewer platform crossings, and reduced time spent in the target quadrant were observed in aged rats and were accompanied by reduced DA, DOPAC, and HVA levels in the CPu and HIPP. Administration of PTX significantly ameliorated these motor and cognitive deficits and increased DA and metabolite levels in aged rats. Similarly, previous studies revealed that PTX improves deficits in learning and memory in β-amyloid protein-induced rats [31] and status epilepticus rats [27]. Our results suggest that PTX administration might help delay aging-related processes in the rat brain.

Accumulation of oxidative damage to macromolecules has been proposed as a mechanism underlying aging and age-related neurodegenerative diseases [38]. ROS such as superoxide anions, hydrogen peroxide (H_2_O_2_), and hydroxyl radicals can cause considerable oxidative damage to proteins, lipids, carbohydrates, and nucleic acids, thereby expediting aging and neurodegeneration [39]. A complex defensive network has evolved to scavenge excess ROS and regulate redox homeostasis [40], and GSH/GSSG ratio is a biomarker of redox status in biological systems. Glutathione peroxidase catalyzes GSH to form GSSG and water, leading to reductions in H_2_O_2_ levels [41]. Lipid peroxidation produces MDA, which is a biomarker of ROS-mediated cell membrane damage [42]. Here, we found that MDA levels were increased and the GSH/GSSG ratio was decreased in the SN and HIPP of aged rats. Reduced MDA levels as well as increased GSH/GSSG ratios after PTX in aged rats demonstrated that PTX exerts antioxidative effects by normalizing oxidant/antioxidant balance in brain tissue and mitochondria during aging [43].

A group of genes and signal transduction pathways that are activated by ROS [44], including the particularly important Nrf2 pathway [40], help to maintain cellular redox balance. Under normal physiological conditions, Nrf2 is inhibited by cytoplasmic kelch-like ECH-associated protein1 (Keap1) and degraded by the proteasome [45]. Under stress conditions, Nrf2 is released from Keap1 and translocates to the nucleus where it binds to ARE, prompting the expression of diverse cytoprotective gene networks [46] to regulate redox processes [14]. Nrf2 also regulates mitochondrial function [47]. Loss of Nrf2 results in mitochondrial depolarization, decreased ATP levels, and impaired respiration, while activation of Nrf2 increases mitochondrial membrane potential, ATP levels, respiration rate, and the efficiency of oxidative phosphorylation [48]. Previous studies revealed that the enzymatic activity of MRC complexes I and IV decreases in the brain, as does ATP synthase (also known as complex V) activity in the SN, during the aging process, while the activity of complexes II and III is largely unaffected [49, 50]. These reductions in complex I, IV, and V activity may decrease ATP production during aging [8]. In the present study, nuclear Nrf2 levels, MRC complex V activity, and mitochondrial ATP levels were reduced in the SN and HIPP, and MRC complex I activity was also reduced in the SN, in 24-month-old aged rats. PTX treatment increased nuclear Nrf2 levels and ameliorated mitochondrial function deficits in aged rats. The reduced complex I activity observed specifically in the SN of aged rats might be associated with the production of ROS as a result of dopamine auto-oxidation in dopaminergic neurons, which are abundant in the SN [51]. This reduced MRC complex I activity in the SN may be particularly sensitive to PTX treatment in aged rats, allowing PTX to reverse aged-related declines in a brain region-specific manner. The increases observed in MRC complex activity, ATP levels, and nuclear Nrf2 levels in aged rats after PTX treatment indicate that Nrf2 activation might mediate PTX-induced delays in brain aging by enhancing antioxidative capability and reversing mitochondrial dysfunction.

In addition to Nrf2, the transcription factor PPARγ has been identified as a key contributor to the cellular antioxidative defense system [52]. As a coactivator of PPARγ, PGC-1α also modulates cellular ROS levels [20, 53], and knockout of PGC-1α increases sensitivity of mouse SN and HIPP neurons to ROS [20]. In contrast, increasing PGC-1α expression protects cultured neural cells from hydrogen peroxide-induced death [20]. *In vitro* studies showed that PGC-1α induces many ROS-detoxifying enzymes, such as glutathione peroxidase 1 and superoxide dismutase 2, upon oxidative challenge [20]. Furthermore, PTX can prevent LPS/CMS-induced downregulation of PGC-1α [54]. In the present study, PTX-induced increases in PGC-1α mRNA and protein levels in aged rats indicated that PTX administration might enhance antioxidative capacity in the SN and HIPP in aged rats partially by upregulating PGC-1α.

In addition to the ROS defense system, mitochondrial biogenesis might be involved in the antiaging effects of PTX in the brain. Mitochondrial biogenesis is important for the preservation of mitochondrial function [55]. The rate of mitochondrial biogenesis is mainly controlled by PGC-1α [56, 57]. PGC-1α induces mitochondrial biogenesis by regulating various transcription factors such as NRF-1, which modulates the expression of genes encoding subunits of the MRC complexes and mitochondrial translational components [58, 59] and upregulates TFAM levels. TFAM directly regulates mitochondrial DNA replication and transcription [57, 60, 61], and mtDNA copy number is frequently used as an indicator of mitochondrial content. CS, a mitochondrial matrix enzyme, also serves as an indicator of mitochondrial content in tissues [62]. The decreases observed here in PGC-1α/NRF-1/TFAM levels, CS activity, mtDNA copy number, and *ATP6* mRNA levels in aged rats indicated that mitochondrial biogenesis was reduced in both the SN and HIPP, while PTX-induced increases in all of those measures indicated that PTX treatment promoted mitochondrial biogenesis in both brain regions during the aging. Previous studies indicate that Nrf2 also regulates mitochondrial biogenesis [63–65]. Activation of Nrf2 promotes mitochondrial biogenesis by upregulating NRF-1 and TFAM in the mouse heart [65], and knockout of Nrf2 decreases mitochondrial biogenesis in skeletal muscle in an age-dependent manner by down-regulating PGC-1α, NRF-1, and TFAM [63]. An *in vitro* study revealed that exposure of N2a cells to interleukin-1β increases protein interactions between Nrf2 and Keap1, reduces binding of Nrf2 to the *Tfam* gene, and inhibits mitochondrial biogenesis [64]. Thus, PTX-induced increases in PGC-1α, nuclear Nrf2, and NRF-1/TFAM levels in the SN and HIPP of aged rats indicates that the ability of PTX to promote mitochondrial biogenesis may be a key mechanism by which it counteracts detrimental effects of aging in the brain.

Some limitations of the current study should be considered when interpreting the results. First, we focused on alterations in cAMP content in the SN and HIPP of PTX-treated aged rats. We found that PTX treatment significantly increased cAMP levels, which is known to counteract cognitive deficits [66], in both areas. However, the molecular mechanisms and pharmacological profile of PTX are complex. PTX also regulates TNF-a-induced complement C3 synthesis and the nitric oxide system and inhibits phosphodiesterase activity; the roles of these mechanisms in PTX-induced effects on the aging brain were not examined. In addition, potential side effects of long-term PTX treatment in aged rats were not investigated. Furthermore, additional *in vivo* and *in vitro* studies employing Nrf2 and PGC-1α knockout models would help to further elucidate the molecular mechanisms underlying the antiaging effects of PTX, which were not comprehensively characterized here.

In conclusion, PTX treatment helps to reverse detrimental changes that accompany aging in the brain. PTX administration improved motor behavior and cognitive deficits, increased brain mesodopaminergic neurochemical content, and ameliorated brain mitochondrial function in aged rats. Moreover, PTX-induced, Nrf2- and PGC-1α-dependent increases in antioxidative capability and mitochondrial biogenesis might underlie the antiaging effects of PTX. Together, these findings suggest potential applications of PTX in delaying the detrimental effects of aging in the brain.

## MATERIALS AND METHODS

### Animals and housing

Male Sprague Dawley rats were supplied by the Experimental Animal Center of Hebei Medical University. Animals were housed under controlled conditions with a 12-hour light-dark diurnal cycle (lights on at 6:00 AM) at 22 ± 2° C. Food and water were available *ad libitum*. All experimental procedures were conducted in accordance with the “Guidelines for the Care and Use of Mammals in Neuroscience and Behavioral Research” and were approved by the Committee of Ethics on Animal Experiments at Hebei Medical University. All efforts were made to minimize animal suffering.

### Experiment 1

Thirty-six rats were used to study the effects of PTX treatment on brain function in aged rats and to determine the optimal PTX dose by analyzing behavioral parameters, DA and metabolite levels, and oxidative balance status parameters. After acclimating for one week, rats were divided into the following six groups: the 6-month-old group (6Mon), the 24-month-old group (24Mon), the 24-month-old PTX 20 mg/kg group (24Mon-Ptx20), the 24-month-old PTX 40 mg/kg group (24Mon-Ptx40), the 24-month-old PTX 60 mg/kg group (24Mon-Ptx60), and the 24-month-old PTX 100 mg/kg group (24Mon-Ptx100). PTX (Sigma) was prepared in saline and administered orally to aged rats beginning at the age of 21 months for 12 weeks (20, 40, 60, or 100 mg/kg per day). Saline was administered once daily to the 6Mon and 24Mon control groups.

### Experiment 2

Fifty-four rats were used to investigate the effects of PTX treatment on mitochondrial function, Nrf2 and mitochondrial biogenesis-related gene expression, and mitochondrial and cAMP content in the aged brain. The rats were divided into the following three groups: 6Mon, 24Mon, and 24Mon-Ptx60. Rats in the 24Mon-Ptx60 group received 60 mg/kg PTX orally once per day beginning at the age of 21 months for 12 weeks, while rats in the 6Mon and 24Mon groups received saline once daily. This PTX dose was chosen based on the results of Experiment 1.

### Open field test

All experiment 1 rats were subjected to the open field test. The open field apparatus consisted of 4 black walls and a white bottom (100 x 100 x 40 cm) placed in a sound-attenuating chamber and illuminated with a 20 lux light. Each rat was individually placed at the center of the arena and its activity was recorded with a digital video camera for 15 min according to the procedure used in our previous study [67]. Three types of behavior, namely vertical activity, horizontal activity, and total path length, were analyzed. The apparatus was cleaned with 70% ethanol after each test to remove odors.

### Morris water maze test

Rats’ cognitive abilities were tested in the water maze during the last six days of PTX treatment. The maze was located in a quiet room and consisted of a circular water tank (180 cm in diameter, 80 cm high) that was partially filled with water (22 ± 2° C). The water was made opaque by adding milk to prevent visualization of the platform. The pool was divided virtually into four equal quadrants. A colorless escape platform (10 cm in diameter) was hidden 1 cm below the surface of the water in a fixed location. Training trials and spatial probe trials were performed as described previously [68]. Escape latency, number of platform crossings, and time spent in the target quadrant were analyzed.

### Sample preparation

Rats were sacrificed by decapitation and brains were quickly removed. Tissue blocks containing the SN (between 3.00 mm and 4.08 mm), CPu (between 8.64 mm and 10.08 mm), and HIPP (between 5.40 mm and 6.08 mm; all measurements rostral to the interaural axis) were dissected on an ice-cold plate using an ophthalmic scalpel and a stereomicroscope. The tissue blocks were either immediately processed for MDA, GSH/GSSG, mitochondrial complex activity, and ATP levels assays or frozen in liquid nitrogen and stored at −80° C for subsequent LC-MS/MS, CS activity, cAMP content, qPCR, or Western blot analysis.

### LC-MS/MS assay

DA, DOPAC, and HVA levels were determined by LC-MS/MS. Tissue blocks containing the CPu or HIPP were weighed and homogenized in 80% acetonitrile containing 0.1% formic acid (5 μL/mg). Homogenates were centrifuged at 14,000 rpm for 10 min at 4° C and supernatants were collected. LC separation was performed on an Agilent 1200 LC system (Agilent, Santa Clara, USA) using a Synergi Fusion-RP C18 column (50mm x 3.0mm, 4μm) provided by Phenomenex. MS/MS detection was carried out using a 3200 QTRAP^TM^ LC-MS/MS System (Applied Biosystems, Foster City, CA, USA). The multiple-reaction monitoring mode was used for quantification. The principal validation parameters of the LC-MS/MS are described in a previous study [27].

### MDA and GSH/GSSG detection

Tissue blocks containing the SN or HIPP were weighed and homogenized in a 10X volume (w/v) of ice-cold 0.1M phosphate buffer, pH 7.4. Supernatants from centrifuged homogenates were used to assess MDA levels (A003-2-2) or GSH/GSSG ratio (A061-2-1) spectrophotometrically according to the detection kit protocol (Nanjing Jiancheng Bioengineering Institute, China).

### Mitochondrial ATP and mitochondrial complex activity assays

Mitochondria were isolated from SN and HIPP tissue blocks from Experiment 2 rats using the Tissue Mitochondria Isolation Kit (Code C3606, Beyotime Institute of Biotechnology, China). Briefly, SN and HIPP tissue blocks were homogenized in ice-cold buffer (10 mM 4-(2-hydroxyethyl)-1-piperazineethanesulfonic acid, pH 7.5, including 200 mM mannitol, 70 mM sucrose, 1.0 mM ethylene glycol tetraacetic acid, and 2.0 mg/mL serum albumin) and centrifuged at 1000 x *g* at 4° C for 10 min. The supernatant was centrifuged again at 3500 x *g* at 4° C for 10 min to collect the mitochondrial pellet.

ATP levels were measured in isolated mitochondria using an ATP colorimetric assay kit following the manufacturer′s instructions (A095-1-1, Nanjing Jiancheng Biotechnology Institute, China). Total mitochondrial protein samples were incubated with the ATP reaction mixture at 37° C for 30 min and detected at 636 nm using a microplate reader (BioTek Instrument Inc., Highland Park, USA).

The enzymatic activities of MCR complexes I, II, III, IV, and V were measured using spectrophotometric assays according to the mitochondrial complex detection kit protocol and were normalized to the total protein amount (μmol/min/g protein). Detection kits for complexes I (A089-1-1), II (A089-2-1), III (A089-3-1), IV (A089-4-1), and V (A089-5-1) were obtained from the Nanjing Jiancheng Institute of Biotechnology (China).

### qPCR analysis

1 μg of total RNA was extracted from SN and HIPP tissue blocks and reverse transcription was performed to generate the first-strand cDNA template. qPCR was then performed with 1 μL of cDNA, 2 μL of each specific primer, and 2× All-in-One^TM^ qPCR Mix (GeneCopoeia Inc., USA) with a final volume of 20 μL. PCR was performed as follows: an initial cycle at 95° C for 10 min, followed by 40 cycles of 95° C for 15 s, 58° C for 20 s, and 72° C for 27 s. PCR products were analyzed using a melting curve to confirm the specificity of amplification. Expression of the *Nrf2*, *PGC-1α, NRF-1,* *TFAM*, and *ATP6* genes was measured. Relative expression was calculated using the 2^−ΔΔCt^ method. Glyceraldehyde-3-phosphate dehydrogenase (*GAPDH*) was used as the reference gene for all calculations. The primer sets were as follows: *Nrf2* (5′-GACCTAAAGCACAGCCAACACAT-3′ and 5′-CTCAATCGGCTTGAATGTTTGTC-3′), *PGC-1α* (5′-AACAGCAAAAGCCACAAAGA-3′ and 5′-AAGTTGTTGGTTTGGCTTGA-3′), *NRF-1* (5′-TCTGCTGTGGCTGATGGAGAGG-3′ and 5′-GATGCTTGCGTCGTCTGGATGG-3′), *TFAM* (5′-TCTCATGATGAAAAGCAGGCA-3′ and 5′-GAGATCACTTCGCCCAACTT-3′), *ATP6* (5′-TACCACTCAGCTATCTATAAACCTAAGCA-3′ and 5′-AGTTTGTGTCGGAAGCCTAGAATT-3′), and *GAPDH* (5′-TGAACGGGAAGCTCACTG-3′ and 5′-GCTTCACCACCTTCTTGATG-3′).

### Western blot analysis

SN and HIPP tissue blocks were homogenized in ice-cold Radioimmunoprecipitation Assay (RIPA) buffer containing 1% Triton X-100, 0.1% sodium dodecyl sulfate (SDS), 0.5% sodium deoxycholate, and protease inhibitors (100 μg/ml phenylmethanesulfonyl fluoride, 30 μg/ml aprotinin, and 1 mM sodium orthovanadate) for 15 min. NP-40 was then added, the homogenate was centrifuged at 10,000 rpm at 4° C for 3 min, and the supernatant containing cytoplasmic protein was collected for detection of PGC-1α, NRF-1, or TFAM. Pellets were homogenized in ice-cold lysis buffer (20 mM HEPES, pH 7.9, 400 mM NaCl, 1 mM EDTA, 0.1 mM EGTA) for 15 min. After centrifugation at 12,000 rpm for 10 min at 4° C, the supernatant was collected. Phenylmethanesulfonyl fluoride was added to the supernatant at a final concentration of 1 mM for detection of nuclear Nrf2 protein. Samples from the SN and HIPP were separated by SDS-PAGE and transferred to polyvinylidene difluoride membranes (Millipore). The membrane was blocked with 5% skimmed milk for 1 h and then incubated overnight with a rabbit anti-Nrf2 polyclonal antibody (1:500, Abcam), rabbit anti-PGC-1α monoclonal antibody (1:1000, Abcam), rabbit anti-NRF-1 polyclonal antibody (1:1000, ABclonal), or rabbit anti-TFAM polyclonal antibody (1:1000, GeneTex) at 4° C. After three washes, the membrane was incubated for 1 h in IRDye® 800-conjugated goat anti-rabbit secondary antibody (1:10000, Rockland) at room temperature. Relative band density was analyzed on an Odyssey infrared scanner (LI-COR Biosciences, USA). Following stripping, each membrane was immunoblotted with anti-histone 3 monoclonal antibody (H3, 1:1000, bioWORLD) or anti-β-actin monoclonal antibody (1:6000, Santa Cruz Biotechnology). Densitometry values were normalized with respect to anti-histone 3 for Nrf2 or anti-β-actin for PGC-1α, NRF-1, and TFAM. Labeling densities were analyzed in Gel-Pro Analyzer Analysis software (Media Cybernetics).

### CS activity assay

Mitochondria were isolated from SN and HIPP tissue blocks using the Minute™ Mitochondria Isolation kit (cat. no. MP-007, Invent Biotechnologies, USA). Isolated mitochondria were dissolved in Minute™ Non-Denatured Protein Solubilization Reagent (cat. no. WA-010, Invent Biotechnologies, USA) and used to assess CS activity. CS activity was measured spectrophotometrically at 412 nm according to the MitoCheck^®^ Citrate Synthase Activity Assay Kit instructions (Item No. 701040, Cayman, USA).

### mtDNA copy number determination

Genomic DNA was extracted from the SN and HIPP tissue blocks using the Animal Tissue Genomic DNA Kit (ZP307-2, Beijing Zoman Biotechnology Institute, China). mtDNA copy number was normalized to the single-copy nuclear *Hbb* (*β-globin*) gene and measured using real-time quantitative PCR (qPCR) and the 2^-ΔΔCt^ method. Primer sequences for the mitochondrial segment were as follows: (5′-CCCTAACACCAGCCTAACCA-3′ and 5′-AAAGTGCATACCGCCAAAAG-3′). Primer sequences for the single-copy nuclear control were as follows: (5′-CTATGGGACGCTTGATGT-3′ and 5′-GCAATCATTCGTCTGTTT-3′).

### cAMP measurement

Tissue blocks containing the SN or HIPP from Experiment 2 rats were homogenized with 50mM glacial acetic acid buffer (4° C, pH 4.75). Supernatants from the centrifuged homogenates were used to assess cAMP content according to the cAMP RIA Kit protocol (HY-10153, Beijing Huaying Biotechnology Institute, China). cAMP content was measured by RIA γ counter and expressed as pmol/mg protein.

### Statistical analysis

Data are expressed as the mean ± standard deviation (S.D.). Grubb’s test was applied to remove possible outliers. The Kolmogorov-Smirnov normality test and Levene’s homogeneity of variance test were applied to all data. If the data were both normally distributed (*P*>0.1) and homogeneous with regard to variance (*P*>0.05), parametric one-way analysis of variance (one-way ANOVA) (F-statistic) tests followed by Tukey’s honestly significant difference (Tukey’s HSD) post hoc tests for multiple comparisons were performed. Otherwise, non-parametric Welch’s F tests for one-way ANOVA (F’-statistic) followed by the Games-Howell procedure for post hoc analysis between groups were performed [69]. Statistical analysis was performed using Statistical Package for the Social Sciences 21 software (SPSS Inc., Chicago, IL, USA) and Prism 8 (GraphPad Software Inc., La Jolla, CA). A *P* value of less than 0.05 was considered statistically significant.
